# Hypoimmunogenic human pluripotent stem cells are valid cell sources for cell therapeutics with normal self-renewal and multilineage differentiation capacity

**DOI:** 10.1186/s13287-022-03233-z

**Published:** 2023-01-24

**Authors:** Yifan Chen, Yanjie Zhou, Zhongshu Zhou, Yujiang Fang, Lin Ma, Xiaoqing Zhang, Jie Xiong, Ling Liu

**Affiliations:** 1 Translational Medical Center for Stem Cell Therapy, Shanghai East Hospital, School of Medicine, Tongji University, Shanghai, China; 2 Key Laboratory of Spine and Spinal Cord Injury Repair and Regeneration of Ministry of Education, Orthopaedic Department of Tongji Hospital, Shanghai, China; 3 Shanghai Institute of Stem Cell Research and Clinical Translation, Shanghai, China; 4 Key Laboratory of Neuroregeneration of Shanghai Universities, School of Medicine, Tongji University, Shanghai, China; 5 Clinical Center for Brain and Spinal Cord Research, Tongji University, Shanghai, China; 6 Tsingtao Advanced Research Institute, Tongji University, Qingdao, China

**Keywords:** Hypoimmunogenic hPSCs, Self-renewal, Multilineage differentiation, Functional maturity

## Abstract

Hypoimmunogenic human pluripotent stem cells (hPSCs) are expected to serve as an unlimited cell source for generating universally compatible “off-the-shelf” cell grafts. However, whether the engineered hypoimmunogenic hPSCs still preserve their advantages of unlimited self-renewal and multilineage differentiation to yield functional tissue cells remains unclear. Here, we systematically studied the self-renewal and differentiation potency of three types of hypoimmunogenic hPSCs, established through the biallelic lesion of B2M gene to remove all surface expression of classical and nonclassical HLA class I molecules (B2M^null^), biallelic homologous recombination of nonclassical HLA-G1 to the B2M loci to knockout B2M while expressing membrane-bound β2m-HLA-G1 fusion proteins (B2M^mHLAG^), and ectopic expression of soluble and secreted β2m-HLA-G5 fusion proteins in B2M^mHLAG^ hPSCs (B2M^m/sHLAG^) in the most widely used WA09 human embryonic stem cells. Our results showed that hypoimmunogenic hPSCs with variable expression patterns of HLA molecules and immune compromising spectrums retained their normal self-renewal capacity and three-germ-layer differentiation potency. More importantly, as exemplified by neurons, cardiomyocytes and hepatocytes, hypoimmunogenic hPSC-derived tissue cells were fully functional as of their morphology, electrophysiological properties, macromolecule transportation and metabolic regulation. Our findings thus indicate that engineered hypoimmunogenic hPSCs hold great promise of serving as an unlimited universal cell source for cell therapeutics.

**Supplementary Information:**

The online version contains supplementary material available at 10.1186/s13287-022-03233-z.

## Introduction

Human pluripotent stem cells (hPSCs), including human embryonic stem cells (hESCs) and human induced pluripotent stem cells (hiPSCs), are valuable cell sources for regenerative strategies as they can generate an unlimited amount of functional progeny cells of interest [1–3]. Although autologous iPSCs-derived midbrain dopaminergic progenitor transplantation for the treatment of Parkinson’s disease has yielded amazing outcomes in Parkinsonian monkeys and even patient, this individualized strategy is laborious, costly, and is only practical for chronic diseases [4–6]. Thus, engineering universal hypoimmunogenic hESCs or hiPSCs for allogeneic cell therapies targeting large patient populations could be more economically feasible and avoid long-term immunosuppressive interventions [7–9].

Immune rejection, an inevitable problem in allogeneic cell therapy, results from the mismatching of human leukocyte antigens (HLAs) on the cell surface between patients and allogeneic transplanted cells. HLA class I molecules, such as HLA-A, -B and -C, which are expressed on almost all nucleated cells and platelets, present intracellularly processed peptides to CD8^+^ cytotoxic T cells and enable elimination of antigen-expressing or virus-infected cells [10]. HLA class I molecule is composed of highly polymorphic heavy chain alpha and β2-microglobulin (B2M). The β2-microglobulin forms a heterodimer with HLA class I proteins and is required for HLA class I expression on the cell surface. Thus, the knockout of the B2M gene restricts an immune response from cytotoxic CD8^+^ T cells by depleting all HLA class I molecules [11–17]. Meanwhile, cytotoxic T lymphocyte antigen 4 (CTLA4) and programmed death ligand-1 (PD-L1) are critical immune inhibitory molecules in maintaining peripheral tolerance by restraining T cell activity. Overexpression of CTLA4-immunoglobulin and PD-L1 in hPSCs has been shown to prevent allogenic immune rejection via blocking T cell checkpoint [18].

Despite the inhibition of T cell response, deletion of B2M activates NK cells due to the absence of nonclassical HLA class I molecules, HLA-E and HLA-G [12, 13]. HLA-E is a ligand for the inhibitory CD94/NGK2A complex expressed in part of NK cells [19–21]. HLA-G, another inhibitory ligand expressed at the maternal–fetal interface during pregnancy, acts as a better candidate to fully overcome rejection responses mediated by NK cells [22–25]. Moreover, ectopic expression of CD47 in hPSCs, a “don’t-eat-me” signal that prevents cells from being engulfed by macrophages, also leads to low immunogenicity [26, 27].

We have previously reported that although knockout of B2M gene in hPSCs (B2M^null^) remarkably ameliorates T cell activation and T cell-mediated cytotoxicity, the deletion of B2M propagates NK cell activation and overrepresentation in allografts. Knock-in of HLA-G1 within the frame of endogenous B2M loci biallelically (B2M^mHLAG^) to express membrane-bound β2m-HLA-G1 fusion proteins while concomitantly ablate-free β2m expression in the hPSCs largely abolishes immune rejections caused by T cells, NK cells and macrophages. Overexpression of soluble and secreted form of β2m-HLA-G5 fusion protein in B2M^mHLAG^ hPSCs (B2M^m/sHLAG^) would construct super hypoimmunogenic hPSCs, ensuring a low immunogenic environment with reduced expression of inflammatory cytokines in the allografts [25]. Of note, HLA molecules are expressed in hPSCs and their expressions moderately increase alongside in vivo development and in vitro differentiation [28–30]. Besides, HLA analog molecules in mice (major histocompatibility complex, MHC) play a role in synaptic plasticity during development [31–36] and B2M knockout mice have increased intestinal iron absorption and iron overload in the liver [37]. This arises an important while unaddressed question about whether the engineered hypoimmunogenic hPSCs still preserve their advantages of unlimited self-renewal and multilineage differentiation to yield functional tissue cells. To address this concern, in this study, we systematically studied the self-renewal capacity and differentiation potency into functional tissue cells of B2M^null^, B2M^mHLAG^ and B2M^m/sHLAG^, three types of hypoimmunogenic hPSCs established in the most widely used WA09 hESCs. Our results show that hypoimmunogenic hPSCs with variable expression patterns of HLA molecules and immune compromising spectrums retain their normal self-renewal capacity and could be efficiently differentiated into functional and mature lineage cells, such as electrophysiologically active neurons, beating cardiomyocytes and albumin-secreting hepatocytes.

## Results

### Hypoimmunogenic hPSCs could be maintained long term in culture and keep three-germ-layer differentiation potency

We constructed hypoimmunogenic hPSCs with different strategies, biallelic lesion of B2M gene to remove all surface expression of classical and nonclassical HLA class I molecules (B2M^null^), biallelic homologous recombination of nonclassical HLA-G1 to the B2M loci to knockout B2M while expressing membrane-bound β2m-HLA-G1 fusion proteins (B2M^mHLAG^), and ectopic expression of soluble and secreted β2m-HLA-G5 fusion proteins in B2M^mHLAG^ hPSCs (B2M^m/sHLAG^). hPSCs constructed with these three strategies have shown robust immunotolerance to T cells and NK cells in both in vitro and in vivo allografts [25].

**Fig. 1:**
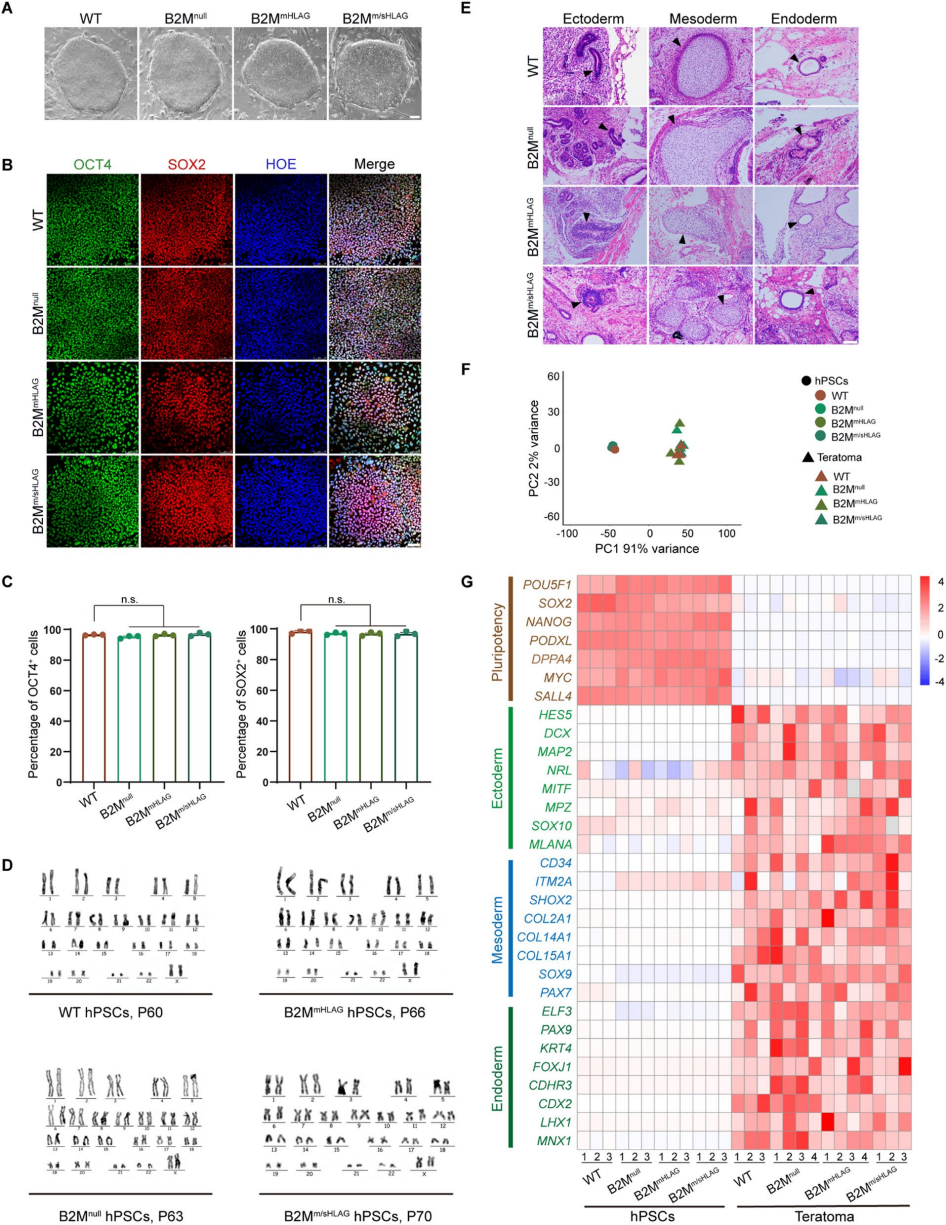
Hypoimmunogenic human pluripotent stem cells (hPSCs) retain normal self-renewal and multilineage differentiation. **A** Bright-field images of wild-type (WT) and hypoimmunogenic hPSCs cultured more than 30 passages. Scale bar, 100 µm. **B** Immunostaining of OCT4 and SOX2 in WT and hypoimmunogenic hPSCs. Nuclei were stained with Hoechst (HOE). Scale bar, 75 µm. **C** FACS quantification showing the percentage of OCT4^+^ and SOX2^+^ cells of WT and hypoimmunogenic hPSCs. Data are presented as mean ± SEM. n.s., no significance, *n* = 3, Student’s *t* test. **D** Karyotype analysis of the WT and hypoimmunogenic hPSCs. **E** H&E staining identified three germ layers in teratomas from WT and hypoimmunogenic hPSCs. Typical tissues were marked by arrowheads (left panel, neural tube-like tissues for ectoderm; middle panel, cartilage-like tissues for mesoderm; right panel, intestine-like tissues for endoderm). Scale bar, 100 μm. **F** Principal component analysis (PCA) plot of WT, hypoimmunogenic hPSCs and teratomas formed by WT and hypoimmunogenic hPSCs. **G** Heatmap of differentially expressed signature genes in RNA-seq data of pluripotency, ectoderm, mesoderm and endoderm in WT, hypoimmunogenic hPSCs and their teratomas

To study whether these engineered hypoimmunogenic hPSCs retain normal self-renewal and differentiation potency, we continuously cultured these cells to more than 30 passages. After passage, all three hypoimmunogenic hPSCs exhibited typical morphology with large nuclear/cytoplasmic ratios, multiple and prominent nucleoli, and round colonies with clear edges resembling of the wild-type (WT) control (Fig. 1A). Immunostaining experiments revealed that all three hypoimmunogenic hPSCs had a uniform nuclear expression of typical pluripotent transcription factors, including OCT4 and SOX2 (Fig. 1B). Flow cytometry analyses further validated that over 95% of cells were OCT4^+^ and SOX2^+^ in long-term cultures (Fig. 1C). G-banding chromosome analysis of hypoimmunogenic hPSCs showed normal karyotype at high passages (Fig. 1D). Moreover, the genetically engineered hPSCs remained correct manipulations in the B2M loci as manifested by genomic PCR analysis, and β2m-HLA-G5 was also highly expressed in long-term cultured B2M^m/sHLAG^ hPSCs (Additional file 1: Fig. S1A, S1B). These data suggest that hypoimmunogenic hPSCs engineered with different strategies retained their pluripotency and could be finely maintained in culture. To transcriptionally characterize the engineered hypoimmunogenic hPSCs, we performed RNA-seq analyses on these cells at passage 55. Dimensionality reduction and clustering by principal component analysis (PCA) demonstrated that hypoimmunogenic hPSCs were clustered with WT hPSCs, but not in vivo differentiated teratomas (Fig. 1E, F). Moreover, similar to the WT control, all three hypoimmunogenic hPSCs showed robust expression of pluripotency genes while lack of expression of three-germ-layer-featured genes (Fig. 1G), highlighting a non-differentiated state of these hypoimmunogenic hPSCs.

To investigate their differentiation potency, three types of hypoimmunogenic hPSCs were subcutaneously injected into NOD scid gamma (NSG) mice, with WT hPSCs as a control, respectively. For all four groups, teratomas were visibly formed at similar occurrence rates after 2 months of injection (Additional file 1: Fig. S1C). Teratomas were then dissected, fixed in paraformaldehyde, followed by hematoxylin–eosin (H&E) staining (Fig. 1E). We observed ectodermal neural tube-like tissues, mesoderm-derived cartilage-like tissues and endodermal intestine-like tissues in WT- and all three hypoimmunogenic hPSCs-injected groups. The dissected teratomas from all groups were also subjected to RNA-seq. Heatmap analyses revealed that teratomas from all three hypoimmunogenic hPSCs and WT hPSCs had similar gene expression patterns featured with all three germ layers (Fig. 1G). Of note, similar to the WT control, we did not observe elevated pluripotent marker genes in any teratomas derived from the three hypoimmunogenic hPSCs. Together, these results suggest that the hypoimmunogenic hPSCs with various HLA expression patterns and immune compromising spectrums could be efficiently differentiated into all three germ layers.

### Neurons differentiated from hypoimmunogenic hPSCs functionally mature and form neural circuits

**Fig. 2:**
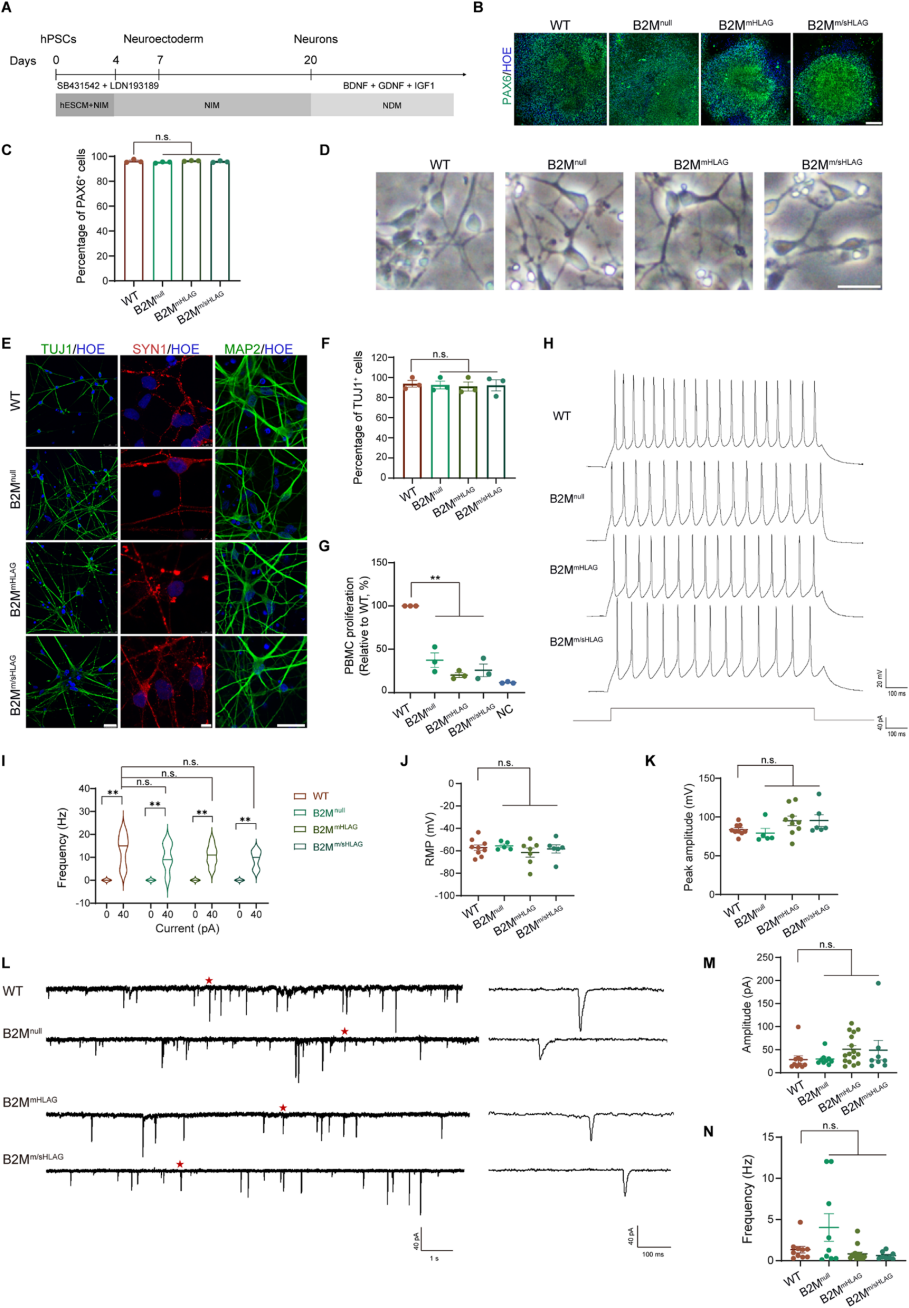
Differentiation of hypoimmunogenic hPSCs into electrophysiologically mature neuronal networks. **A** Schematic of the protocol and stages for neural differentiation of hPSCs. **B** Representative neural tube-like rosette structures formed by PAX6^+^ neuroepithelial cells. Scale bar, 100 μm. **C** FACS quantification showing the percentage of PAX6^+^ cells on day 12 neural differentiation of WT and hypoimmunogenic hPSCs. Data are presented as mean ± SEM. n.s., no significance, *n* = 3, Student’s *t* test. **D** Bright-field images showing neuron morphology derived from WT and hypoimmunogenic hPSCs. Scale bar, 25 μm. **E** Representative immunofluorescence images of TUJ1, SYN1 and MAP2 in WT and hypoimmunogenic hPSCs-derived neurons. Scale bars, left, 25 µm; middle, 5 µm; right, 25 µm. **F** Quantification of the percentage of TUJ1^+^ cells in E. Data are presented as mean ± SEM. n.s., no significance; *n* = 3, Student’s *t* test. **G** MLR analysis showing relative proliferation of PBMCs after coculturing with neurons differentiated from WT, B2M^null^, B2M^mHLAG^, and B2M^m/sHLAG^ hPSCs. PBMCs cultured only were used as a negative control (NC). Data are presented as mean ± SEM. **p < 0.01, *n* = 3, Student’s *t* test. **H** Representative traces from neurons derived from hypoimmunogenic and WT hPSCs firing repetitive APs during depolarizing constant-current injections. Current step is shown in the bottom panel. **I** Violin plots of frequency–current (F-I) among firing neurons (current injection = 0 and 40 pA). Data are presented as mean ± SEM. ** *p* < 0.01, n.s., no significance, *n* (WT) = 8, *n* (B2M^null^) = 5, *n* (B2M^mHLAG^) = 11, *n* (B2M^m/sHLAG^) = 6, Student’s *t* test. **J**, **K** Resting membrane potentials (**J**) and peak amplitudes (**K**) of neurons derived from hypoimmunogenic and WT hPSCs. Parameters were calculated from the first evoked spike. Data are presented as mean ± SEM. For resting membrane potentials, *n* (WT) = 9, *n* (B2M^null^) = 5, *n* (B2M^mHLAG^) = 7, *n* (B2M^m/sHLAG^) = 6. For peak amplitudes, *n* (WT) = 9, *n* (B2M^null^) = 5, *n* (B2M^mHLAG^) = 9, *n* (B2M^m/sHLAG^) = 6. n.s., no significance, Student’s *t* test. **L** Left, representative voltage-clamp recording from neurons derived from hypoimmunogenic and WT hPSCs with spontaneous synaptic input (Vm = −70 mV). Right, zoom-in of the region in left marked by the red five-pointed star, containing one postsynaptic event. **M**, **N** Amplitudes (**M**) and frequencies (**N**) of spontaneous postsynaptic currents of neurons derived from hypoimmunogenic and WT hPSCs. Data are represented as mean ± SEM. n.s., no significance, *n* (WT) = 10, *n* (B2M^null^) = 9, *n* (B2M^mHLAG^) = 16, *n* (B2M^m/sHLAG^) = 8, Student’s *t* test

To study whether hypoimmunogenic hPSCs could be efficiently specified into functional tissue cells, we first differentiated WT hPSCs and all three hypoimmunogenic hPSCs toward cortical neurons via the Dual-Smad inhibition differentiation protocol as previously described [38–41] (Fig. 2A). Neural ectoderm (NE) cells appeared on post-differentiation day 7 and they formed typical neural tube-like rosettes on day 10 in all four groups. Immunostaining experiments showed that on day 10, most NE cells were positive for PAX6 (Fig. 2B), indicating synchronized neural induction in control and hypoimmunogenic hPSC groups. Quantification of the percentage of PAX6^+^ cells in NE cultures further strengthened the conclusion that hypoimmunogenic hPSCs were almost uniformly specified into the PAX6^+^ NE (Fig. 2C).

The specified NE were further differentiated into neural precursors and then to neurons (Fig. 2A). Hypoimmunogenic hPSCs-derived neurons have typical morphology with somas and projections after being plated onto a laminin-coated surface for 3 days (Fig. 2D). On post-differentiation day 30, neurons yielded from all three hypoimmunogenic hPSCs were positive for TUJ1 with extended long projections (Fig. 2E). Quantification of TUJ1^+^ cells showed no significant differences between hypoimmunogenic hPSCs and WT (Fig. 2F). Moreover, MAP2, a more mature neuronal marker, was also highly expressed in neurons derived from all groups (Fig. 2E). The differentiated neurons from hypoimmunogenic hPSCs also expressed punctated synapsin1 (SYN1), a hallmark protein of presynaptic membrane, at 8 weeks post-differentiation (Fig. 2E), indicating gradual synaptic maturation of WT and hypoimmunogenic hPSC-derived neurons.

To assess the immunogenicity of neurons derived from WT and three hypoimmunogenic hPSCs, we performed mixed lymphocyte reactions (MLRs) as previously reported [25]. Neurons were cocultured with peripheral blood mononuclear cells (PBMCs) collected from healthy donors. The HLA alleles of PBMCs from each individual were tested to ensure HLA mismatch between target neurons and effector PBMCs (Additional file 1: Fig. S2A). Compared with WT control, proliferation of PBMCs decreased significantly when cocultured with hypoimmunogenic neurons (Fig. 2G, Additional file 1: Fig. S2B), indicating that neurons derived from B2M^null^, B2M^mHLAG^, and B2M^m/sHLAG^ hPSCs are hypoimmunogenic to immune cells within the PBMCs.

To characterize the functional maturity of the three hypoimmunogenic hPSC-derived neurons, whole-cell patch-clamp electrophysiological recording experiments were performed. All three hypoimmunogenic hPSC-derived neurons had the ability to fire action potentials (APs) repetitively in response to current injection (Fig. 2H, I). AP properties were then quantified and compared to evaluate the electrophysiological maturity of the neurons. The resting membrane potentials (RMPs) of WT-, B2M^null^-, B2M^mHLAG^- and B2M^m/sHLAG^-derived neurons were − 57.18 ± 2.510 mV, − 55.50 ± 1.564 mV, − 61.58 ± 4.026 mV, and − 58.27 ± 3.627 mV, respectively (Fig. 2J). The peak amplitudes were 83.70 ± 2.386 mV, 79.26 ± 6.053 mV, 95.16 ± 6.068 mV, and 95.50 ± 7.588 mV, respectively (Fig. 2K). No significant differences were observed in all four groups in these AP parameters, indicating that the hypoimmunogenic hPSC-derived neurons attained the capacity to fire trains-of-action potentials by 8–10 weeks, the same time point for neurons derived from the WT hPSCs. In addition, there were no differences in synaptic connectivity amongst the WT and hypoimmunogenic hPSC-derived neurons (Fig. 2L–N). The amplitudes of spontaneous synaptic activity were 28.46 ± 8.173 pA, 29.63 ± 4.473 pA, 50.98 ± 7.815 pA, and 48.94 ± 21.18 pA, respectively (Fig. 2M). The frequencies of spontaneous synaptic activity from WT-, B2M^null^-, B2M^mHLAG^- and B2M^m/sHLAG^-derived neurons were 1.33 ± 0.4024 Hz, 4.032 ± 1.676 Hz, 0.8269 ± 0.2159 Hz, and 0.6134 ± 0.1601 Hz, respectively (Fig. 2N). Taken together, these data suggest that the three types of hypoimmunogenic hPSCs are normally differentiated into electrophysiologically mature neurons in culture.

### Hypoimmunogenic hPSC-derived cardiomyocytes spontaneously contract and possess functionally electrophysiological characteristics

**Fig. 3:**
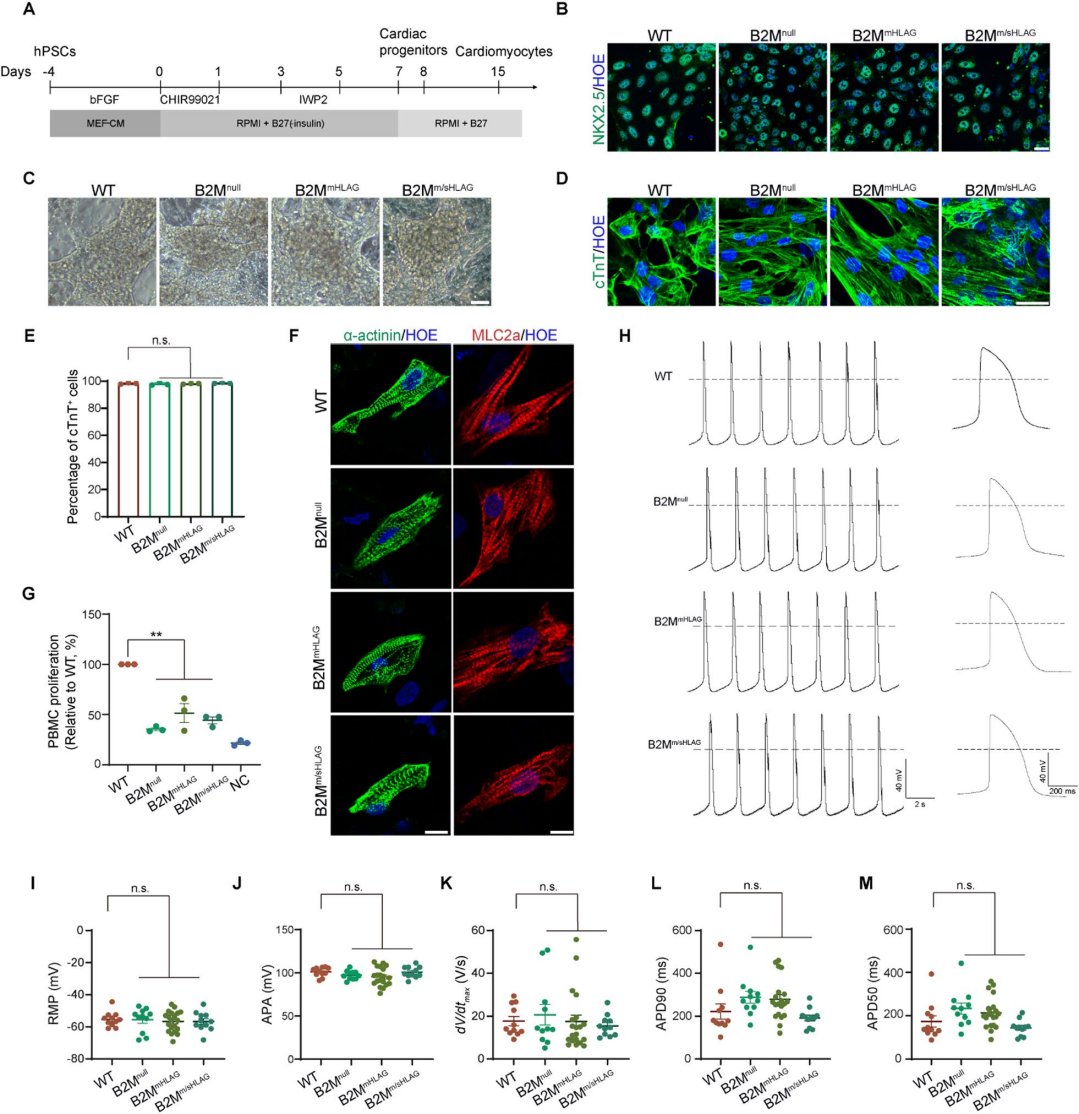
Characterization of cardiomyocytes generated from hypoimmunogenic hPSCs. **A** Schematic of the protocol and stages for the cardiac differentiation of hPSCs. **B** Representative immunofluorescence images of NKX2.5 on day 8 of cardiac differentiation of WT and hypoimmunogenic hPSCs. Scale bar, 25 μm. **C** Bright-field images showing cardiomyocytes morphology derived from WT and hypoimmunogenic hPSCs. Scale bar, 50 μm. **D** Representative immunofluorescence images of cTnT at 3 weeks of cardiac differentiation. Scale bar, 25 μm. **E** FACS quantification showing percentages of differentiated cTnT^+^ cardiomyocyte-like cells. Data are represented as mean ± SEM. n.s., no significance, *n* = 3, Student’s *t* test. **F** Representative immunofluorescence images of sarcomere organization by α-actinin and MLC2a staining. Scale bars, 25 μm. **G** MLR analysis showing relative proliferation of PBMCs after coculturing with cardiomyocytes differentiated from WT, B2M^null^, B2M^mHLAG^, and B2M^m/sHLAG^ hPSCs. PBMCs cultured only were used as a negative control (NC). Data are presented as mean ± SEM. **p < 0.01, *n* = 3, Student’s *t* test. **H** Representative recordings of ventricular-like action potentials using whole-cell patch clamp from cardiomyocytes differentiated from WT and hypoimmunogenic hPSCs. Dotted line indicates 0 mV. Right, single action potential at an expanded timescale taken from traces on the left. **I**–**M** Comparison of rest membrane potential (RMP, **I**), action potential amplitude (APA, **J**), maximal rate of depolarization (*d*V/*d*t_max_, **K**), action potential duration (APD) at 90% repolarization (**L**) and 50% repolarization (**M**) in cardiomyocytes differentiated from WT and hypoimmunogenic hPSCs. Data are represented as mean ± SEM. n.s., no significance, *n* (WT) = 11, *n* (B2M^null^) = 11, *n* (B2M^mHLAG^) = 20, *n* (B2M^m/sHLAG^) = 11, Student’s *t* test

Cardiomyocyte transplantation has been considered as a replacement for heart transplantation and conventional regenerative therapies [42–44]. To study whether hypoimmunogenic hPSCs hold the ability to differentiate into functional and mature cardiomyocytes, WT, B2M^null^, B2M^mHLAG^ and B2M^m/sHLAG^ hPSCs were differentiated toward a cardiomyocyte fate as previously described [45, 46] (Fig. 3A). On day 8, cells differentiated from WT and all three hypoimmunogenic hPSCs were uniformly positive for NKX2.5, a cardiac transcription factor, suggesting synchronized cardiac fate specification (Fig. 3B). On day 12, cardiomyocytes derived from hypoimmunogenic hPSCs as well as WT hPSCs began to spontaneously contract, and these cardiomyocytes beat robustly even after 100 days of differentiation (Fig. 3C, Additional file 2: video S1, Additional file 3: video S2, Additional file 4: video S3, Additional file 5: video S4). The hypoimmunogenic hPSC-derived cardiomyocytes were also positive for cardiac troponin T (cTnT), a highly cardiac-specific myofilament protein. Quantification studies revealed that the percentage of cTnT^+^ cardiomyocytes in the entire culture of WT and all three hypoimmunogenic hPSCs were over 95% with batch-to-batch consistency (Fig. 3D, E). To evaluate the cardiac sarcomere organization, cells were labeled with α-actinin, the Z-line marker of the sarcomere, and myosin light chain 2 atrial isoform (MLC2a), the A-band marker of the sarcomere. Again, hypoimmunogenic hPSC-derived cardiomyocytes showed typical α-actinin and MLC2a staining (Fig. 3F). Taken together, immunolabeling of multiple myofilament proteins indicates that a well-organized sarcomeric structure can be similarly developed in all three hypoimmunogenic hPSC-derived cardiomyocytes. MLRs studies confirmed that cardiomyocytes derived from B2M^null^, B2M^mHLAG^, and B2M^m/sHLAG^ hPSCs led to a much lower proliferation of cocultured PBMCs and again suggested their hypoimmunogenicity (Fig. 3G, Additional file 1: Fig. S2B).

To assess the maturity of cardiomyocytes derived from hypoimmunogenic hPSCs, we performed electrophysiological studies in cardiomyocytes derived from WT, B2M^null^, B2M^mHLAG^ and B2M^m/sHLAG^ hPSCs 30–35 days post-differentiation, a time window when ventricular-like cells being the predominant phenotype [47]. A majority of hypoimmunogenic hPSC-derived cardiomyocytes exhibited spontaneous ventricular-like electrical activity, similarly to Burridge’s report [47]. Representative recordings of ventricular-like action potentials are shown in Fig. 3H. Specifically, the RMPs of WT-, B2M^null^-, B2M^mHLAG^- and B2M^m/sHLAG^-derived ventricular-like cells were − 55.30 ± 1.398 mV, − 55.60 ± 2.230 mV, − 56.64 ± 1.421 mV and − 56.65 ± 1.779 mV, respectively (Fig. 3I). The action potential amplitudes (APAs) of WT-, B2M^null^-, B2M^mHLAG^- and B2M^m/sHLAG^-derived ventricular-like cells were 101.0 ± 1.610 mV, 97.39 ± 1.530 mV, 95.52 ± 2.343 mV, and 100.9 ± 1.935 mV, respectively (Fig. 3J). The maximal rates of depolarization (d*V/*d*t*_max_) of WT-, B2M^null^-, B2M^mHLAG^- and B2M^m/sHLAG^-derived ventricular-like cells were 17.70 ± 2.163 V/s, 20.70 ± 4.778 V/s, 17.46 ± 3.062 V/s, and 15.46 ± 1.509 V/s, respectively (Fig. 3K). The action potential durations (APDs) at different levels of repolarization (90% and 50%, APD90 and APD50) of WT-, B2M^null^-, B2M^mHLAG^- and B2M^m/sHLAG^-derived ventricular-like cells were 221.6 ± 35.50 ms and 173.7 ± 25.68 ms, 288.1 ± 27.89 ms and 233.2 ± 25.85 ms, 278.9 ± 21.61 ms and 213.3 ± 16.27 ms, and 191.7 ± 13.72 and 144.0 ± 11.20 ms, respectively (Fig. 3L, M). Quantification data of the AP properties of ventricular cells derived from each group were presented, and they showed no significant differences among groups. These data suggest that the engineered hypoimmunogenic hPSCs by modifying HLA class I molecules could be faithfully differentiated into functionally mature cardiomyocytes with proper cytoskeleton morphology and electrophysiological activities.

### Hypoimmunogenic hPSCs differentiate into hepatocytes with featured metabolic functions

**Fig. 4:**
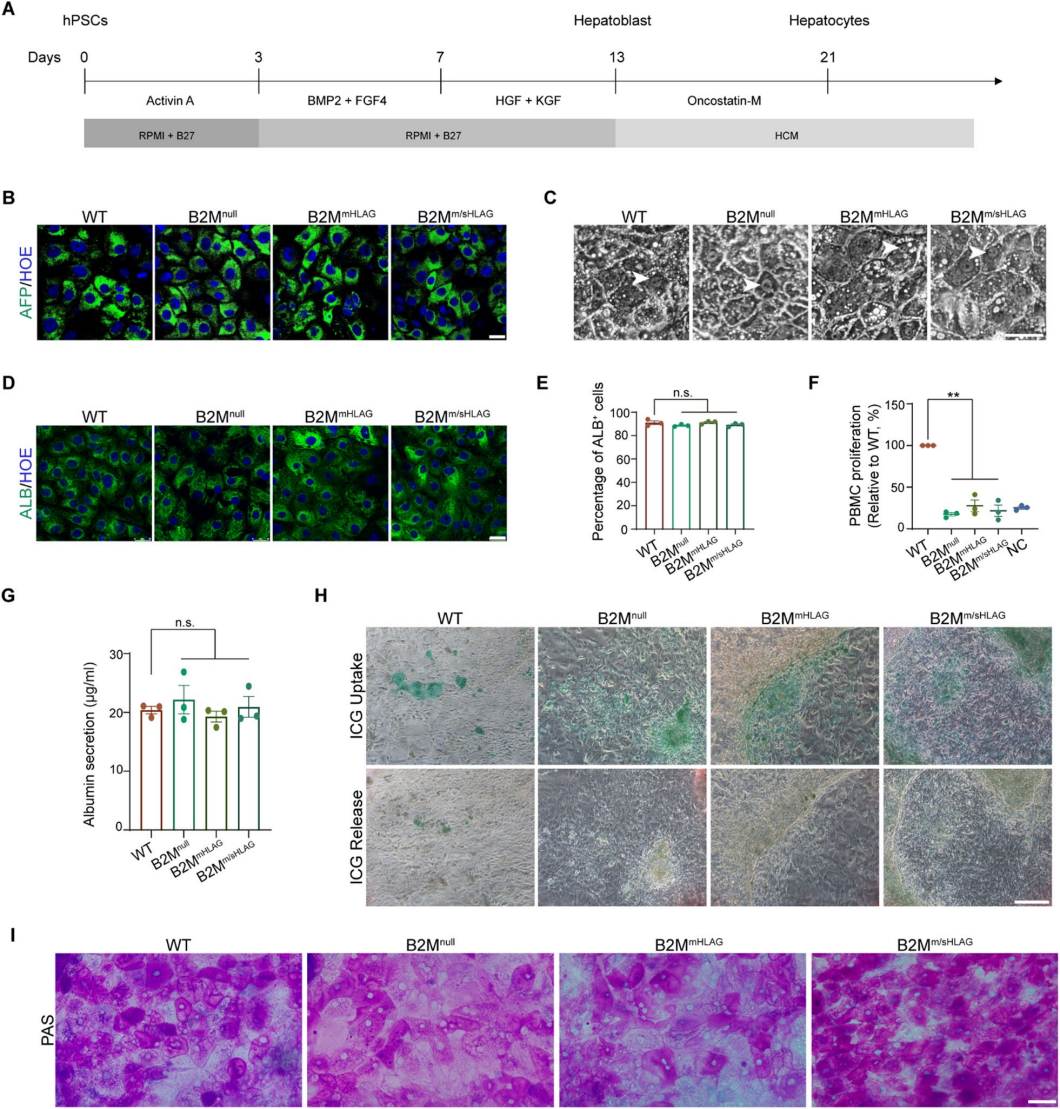
Functional analysis of the hepatocytes derived from WT and hypoimmunogenic hPSCs. **A** Schematic of the protocol and stages for the hepatic differentiation of hPSCs. **B** Representative immunofluorescence images of alpha-fetoprotein (AFP) in hepatocytes differentiated from WT and hypoimmunogenic hPSCs. Scale bar, 25 μm. **C** Bright-field images showing hepatocytes morphology derived from WT and hypoimmunogenic hPSCs. Arrowheads, binucleated hepatocytes. Scale bar, 50 μm. **D** Representative immunofluorescence images of albumin (ALB) in hepatocytes differentiated from WT and hypoimmunogenic hPSCs. Scale bar, 25 μm. **E** FACS quantification showing the percentages of ALB^+^ cells on day 21 hepatic differentiation of WT and hypoimmunogenic hPSCs. Data are represented as mean ± SEM. n.s., no significance, *n* = 3, Student’s *t* test. **F** MLR analysis showing relative proliferation of PBMCs after coculturing with hepatocytes differentiated from WT, B2M^null^, B2M^mHLAG^, and B2M^m/sHLAG^ hPSCs. PBMCs cultured only were used as a negative control (NC). Data are presented as mean ± SEM. **p < 0.01, *n* = 3, Student’s *t* test. **G** ELISA quantification showing ALB secretion from WT- and hypoimmunogenic hPSCs-derived hepatocytes. Data are represented as mean ± SEM. n.s., no significance, *n* = 3, Student’s *t* test. **H** ICG uptake and release assay of WT- and hypoimmunogenic hPSCs-derived hepatocytes. Scale bar, 250 μm. **I** Glycogen synthesis analysis by PAS staining on day 21 hepatic differentiation of WT and hypoimmunogenic hPSCs. Scale bar, 50 μm

Various neurons and cardiomyocytes, which are typical for their induced or spontaneous electrophysiological excitability, and hepatocytes have more complex cellular functions related to metabolic pathways, such as cargo transport, insulin-regulated glucose metabolism, and detoxification. We used a four-step protocol to drive hPSCs toward a definitive endoderm and then a hepatocyte fate [48, 49] (Fig. 4A). Immunostaining experiments revealed that the vast majority of the differentiation derivatives from WT and all three hypoimmunogenic hPSCs were positive for alpha-fetoprotein (AFP) on day 13 (Fig. 4B), suggesting a uniform hepatic precursor fate (HPCs) obtained in all cultures. On day 21, differentiated cells exhibited the characteristic morphology of primary hepatocytes, including a large cytoplasmic-to-nuclear ratio, numerous vacuoles and vesicles, and prominent nucleoli (Fig. 4C). Binucleated cells were also observed (Fig. 4C). Albumin (ALB), a marker of mature hepatocytes, was detected in hepatocyte-like cells (Fig. 4D). FACS analysis revealed that ~ 90% cells were positive for ALB in WT and all three hypoimmunogenic hPSCs cultures, and they preserved their hypoimmunogenicity after hepatocyte differentiation (Fig. 4E, F). These results suggest that the engineered hPSCs are successfully specified into hypoimmunogenic hepatocytes with high efficiency.

ALB synthesis assays were further performed to specifically test the metabolic activities of differentiated hepatocytes. The concentrations of secreted ALB in the supernatants of day 21 hepatocytes cultures from WT, B2M^null^, B2M^mHLAG^ and B2M^m/sHLAG^ hPSCs were 20.40 ± 0.631 μg/mL, 22.18 ± 2.424 μg/mL, 19.31 ± 0.927 μg/mL, and 20.95 ± 1.763 μg/mL, respectively, with no obvious differences within each group (Fig. 4G). Indocyanine green (ICG) uptake and release assays showed that all three hypoimmunogenic hPSC-derived hepatocytes exhibited clear ICG uptake and release within 6 h similarly to the WT control (Fig. 4H). In addition, periodic acid Schiff (PAS) staining revealed comparable glycogen storage in all four groups (Fig. 4I). These results indicate that functional hepatocyte-like cells can be derived from hypoimmunogenic hPSCs, and these hypoimmunogenic hPSCs-derived cells have regular metabolic functions, such as macromolecule transportation and glucose metabolism.

## Discussion

In this study, we explored the self-renewal capacity and the functionality of their differentiated tissue cells of hypoimmunogenic hPSCs with different HLA class I presentation patterns. The B2M^null^ hPSCs lack surface expression of classical and nonclassical HLA class I molecules. The B2M^mHLAG^ hPSCs and the B2M^m/sHLAG^ hPSCs lack cell surface expression of classical HLA class I molecules, but have membrane-bound β2m-HLA-G1 fusion proteins expression, and the B2M^m/sHLAG^ hPSCs also ectopically express soluble and secreted β2m-HLA-G5. We present here promising evidence and show that all three hypoimmunogenic hPSCs retain their normal self-renewal capacity and could be efficiently differentiated into functional and mature lineage cells, including electrophysiologically active neurons, beating cardiomyocytes and albumin-secreting hepatocytes. To our knowledge, this is the first research to systematically study and address the question of whether the engineered hypoimmunogenic hPSCs with variable expression patterns of HLA molecules and immune compromising spectrums still preserve their advantages of unlimited self-renewal and multilineage differentiation to yield functional tissue cells, which paves the way for future applications of these engineered cells for cell therapeutics.

Here, we reveal that the B2M^null^, B2M^mHLAG^ and B2M^m/sHLAG^ hPSCs have typical morphology and can be long-term maintained in culture with no differences observed from that of the WT hPSCs. RNA-seq analyses show that hypoimmunogenic hPSCs and WT hPSCs have similar gene expression profiles. Meanwhile, these hypoimmunogenic hPSCs could be targeted to ectodermal, mesodermal and endodermal lineages, as exemplified by neural precursors, cardiac precursors and hepatic precursors, with high efficiency. Teratomas yielded from hypoimmunogenic hPSCs and WT hPSCs harbor lineages of all three germ layers, and transcriptional profiling studies elucidate that they are comparable to the upregulation of featured genes related to all three germ layers and downregulation of genes related to pluripotency. These results firmly secure the conclusion that all three hypoimmunogenic hPSCs are phenotypically normal in pluripotency maintenance and three-germ-layer differentiation. It is reported that classical HLA class I molecules are expressed in hPSCs and their expression moderately increases alongside in vivo development and in vitro differentiation [28–30]. Given knockout of B2M does not interfere with hPSCs self-renewal or three germ-layer differentiation, this indicates that expression of HLA class I molecules on the surface of hPSCs is not intrinsically required for their function. HLA-G is not present in hPSCs [28]. Here, we show that ectopic expression of either membrane-bound or secreted HLA-G in hPSCs has no effect on their normal self-renewal and three germ-layer differentiation capacity, coinciding with their early physiological expression at the fetomaternal interface in cytotrophoblasts during embryonic development [22, 23].

Mice with B2M knockout exhibit aberrant axonal and dendritic outgrowth, abnormal synapse density and expanded ipsilateral projection [36, 50–52]. In our current study, hPSCs with B2M knockout are regularly differentiated into neurons with no observed aberrant synaptic density and neurite outgrowth. Whole-cell patch clamping further reveals that B2M knockout hPSC-derived neurons attain the normal capacity to fire trains-of-action potentials and display spontaneous postsynaptic currents in vitro. It is therefore reasonable to conclude that hypoimmunogenic hPSCs hold normal intrinsic programs to differentiate into morphologically and electrophysiologically mature neurons and these neurons could organize into functional neural circuits. In the in vivo context, lack of surface expression of MHC molecules expression in cell types other than neurons, such as astrocytes or microglia, might lead to a malfunction of the immune milieu, which subsequently causes aberrant neuronal development, maturation and neuronal transmission. Indeed, MHC molecules expression in microglia is required for synaptic development [53]. On the other hand, B2M-deficient mice fail to express MHCI molecules, resulting in systemic iron overload and consequently hepatocytes necrosis and abnormal metabolic function [54]. Here, the hypoimmunogenic hPSC-derived hepatocytes are phenotypically normal and possess natural metabolic functions. This again supports the hypothesis that the expression patterns of MHC molecules in hepatocytes might be not essential for their normal function. Of note, ectopic expression of membrane-bound or soluble HLA-G proteins could ameliorate abnormal overactivation of NK cells and antigen-presenting cells, which could serve as a pledge of supporting normal development and function of derivatives of these hypoimmunogenic hPSCs after allograft transplantations [25]. In the future, in vivo transplantation studies in animal models or clinical trials will be expected in order to propagate our current study to apply these universal cell sources for cell therapeutics.

Although all three types of hypoimmunogenic hPSCs and their derivatives cannot be recognized by CD8^+^ T cells, a complete lack of surface expression of classical HLA class I molecules in B2M^null^ hPSCs would activate NK cells due to missing-self response [25]. B2M^mHLAG^ and B2M^m/sHLAG^ hPSCs have no surface expression of classical HLA class I molecules, but have an ectopic expression of HLA-G proteins instead, and they preserve their capability in compromising T cell activation and are resistant to immune attack mediated by NK cells [25]. For therapeutic purpose, B2M^mHLAG^ and B2M^m/sHLAG^ hPSCs would be more ideal in order to obtain a wider hypoimmunogenic spectrum. Meanwhile, B2M^m/sHLAG^ hPSCs secret β2m-HLA-G protein and could safeguard a low immunogenic environment after transplantation. Therefore, B2M^m/sHLAG^ hPSCs and their derivatives are super hypoimmunogenic, and they are optimal for cell transplantations in non-immunologically privileged organs or for diseases with proinflammatory cytokines. It is also worth noting that, for a clinical use purpose, the compliance with current good manufacturing practice (cGMP) protocols in culturing and engineering either hESCs or hiPSCs in feeder-free, xeno-free and chemically defined conditions is obviously required for future studies.

## Conclusions

In the current study, we present evidence and show that hypoimmunogenic hPSCs with variable expression patterns of HLA molecules and immune compromising spectrums retain their normal self-renewal capacity and could be efficiently differentiated into functional and mature lineage cells, such as electrophysiologically active neurons, beating cardiomyocytes and albumin-secreting hepatocytes. These hypoimmunogenic hPSCs hold great promise to serve as unlimited universal cell sources for cell therapeutics.

## Materials and methods

### Cell culture and differentiation

Undifferentiated human H9 ES cells (WA09, WiCell agreement no. 14-W0377) and hypoimmunogenic hPSCs established in WA09 hESCs were maintained on irradiated mouse embryonic fibroblasts (MEF) in hESCM containing DMEM/F12, 20% knockout serum replacer, 1 × non-essential amino acids, 1 × GlutaMAX, and 0.1 mM β-mercaptoethanol at 37 °C with 5% CO_2_ as previously described [55–59]. Fibroblast growth factor 2 (FGF2, 4 ng/mL, PeproTech) was added when refreshing medium. Cells were manually passaged at 1:6 split ratio every 5 days with dispase digestion.

For neural differentiation, we used previously described protocols [38–40]. In brief, cells were adherently cultured in hESCM: neural induction medium (NIM) (1:1) for 3 days supplemented with 2 μM SB431542 (Stemgent) and 200 nM LDN193189 (Stemgent), followed by 4 days with 2 μM SB431542 and 200 nM LDN193189 in NIM, then 5 days in NIM. Cells were then lifted and maintained in suspension culture on day 12, then 8 days in NIM. On day 20, cells were plated on laminin-coated coverslips for generating neurons in neural differentiation medium (NDM) with 10 ng/mL BDNF (PeproTech), 10 ng/mL GDNF (PeproTech) and 10 ng/mL IGF (PeproTech). NIM consists of DMEM/F12: neurobasal medium (1:1), N2 supplement and B27 (complete with Insulin). NDM consists of neurobasal medium, N2 supplement and B27 (complete with Insulin), 200 μM ascorbic acid, and 1 μM cAMP.

For cardiomyocyte differentiation, procedures were described previously [45, 46]. In brief, cells were cultured in hESCM from day − 4 to day − 1. Cells were switched to RPMI/B27 medium (Insulin minus, Gibco) supplemented with 12 μM CHIR99021 (Selleck) for 1 day. CHIR99021 was removed from the medium on day 1, and the culture medium was switched to 5 μM IWP2 in RPMI/B27 without insulin on day 3. On day 5 of differentiation, aspirate the medium and add RPMI/B27 without insulin. On day 7 of differentiation and every 3 days thereafter, aspirate the medium and add RPMI/B27 medium.

For hepatic differentiation, cells were cultured based on previously described protocols with minor modifications [48, 49]. Briefly, cells were maintained in hESCM from day − 3 to day 0 and then switched to RPMI/B27 medium (Insulin minus, Gibco) supplemented with 100 ng/mL Activin A (PeproTech) for 3 days. On day 4, cells were switched to RPMI/B27 (complete with Insulin, Gibco) medium supplemented with 20 ng/mL BMP2 (PeproTech) and 30 ng/mL FGF4 (PeproTech) for 4 days. After BMP2 and FGF4 removal, 20 ng/mL HGF (PeproTech) and KGF (PeproTech) were added for 6 days. Afterward, cells were switched to hepatocyte culture media (Lonza) supplemented with 20 ng/mL Oncostatin-M (R&D Systems) and SingleQuots (without EGF) for 8 days.

### RNA-seq library construction and sequencing

Total RNA was extracted using TRIzol reagent (Thermo Fisher Scientific). RNA-seq libraries were constructed using NEBNext Ultra RNA Library Prep Kit for Illumina (NEB). Libraries were sequenced using an Illumina HiSeq X-ten.

### RNA-seq data processing

RNA-seq data were subjected to quality control analysis using FastQC (v0.11.9). All RNA-seq data were mapped to hg38 reference genome using Bowtie2 (v2.4.4) with default parameters. Gene expression levels were normalized using transcripts per million (TPM). Gene expression profiles of different samples were displayed as heatmap; the TPM values were row-scaled for better visualization. Principle component analysis (PCA) was performed with R package ggpolot2.

### Electrophysiology

Hypoimmunogenic hPSC- and hESC-derived neurons were recorded at 8–10 weeks with whole-cell patch clamp at room temperature using a MultiClamp 700B amplifier (Molecular Devices, Sunnyvale, CA, USA). Recording micropipettes (tip resistance 3–6 MΩ) were filled with internal solution composed of (in mM): 140 K-gluconate, 1 EGTA, 2 MgCl_2_, 4 MgATP, 0.3 NaGTP, 10 HEPES and 0.1 CaCl_2_ (pH 7.4). The bath was perfused with artificial cerebrospinal fluid (ACSF) composed of (in mM): 119 NaCl, 1.8 KCl, 2.4 CaCl_2_, 10 glucose, 1 NaH_2_PO_4_, 26.2 NaHCO_3_ and 1.2 MgCl_2_ (pH 7.4). For voltage-clamp recordings, cells were clamped at − 70 mV [60]. Spontaneous postsynaptic currents were recorded for at least 3 min. For current-clamp recordings, voltage responses were evoked from − 10 to + 80 pA in 10 pA intervals. Single action potential (AP) properties were calculated from the first evoked AP in response to a depolarizing step. Quantitative analysis was performed using Clampfit software (Molecular Devices, SanJose, CA) and Mini Analysis Software (Synaptosoft). For cardiomyocytes, cellular action potentials were recorded at day 30–35. Recording micropipettes (tip resistance 3–6 MΩ) were filled with internal solution composed of (in mM): 10 EGTA, 1 MgCl_2_, 3 MgATP, 10 HEPES and 120 KCl (pH 7.2). The bath was perfused with Tyrode’s solution composed of (in mM): 135 NaCl, 5.4 KCl, 1.8 CaCl_2_, 10 glucose and 0.3 Na_2_HPO_4_, 0.3 KH_2_PO_4_ and 10 HEPES (pH 7.35). Quantitative analysis was performed using Clampfit software. The criteria used for classifying observed APs into ventricular, atrial and nodal-like cells were previously described [61]. Cells with ventricular-like action potentials typically displayed a more negative maximum diastolic potential, a rapid action potential upstroke, and a distinct plateau phase. Atrial-like cells were distinguished from ventricular-like cells by the absence of a distinct plateau during repolarization but typically exhibited spontaneous activity that was higher in frequency than that observed in ventricular cells. Nodal-like cells were distinguished by maximum diastolic potentials that were less negative than those of ventricular- and atrial-like cells, smaller amplitude action potentials, characterized as a slower action potential upstroke, and a pronounced phase 4 depolarization preceding the action potential upstroke.

### Immunofluorescence

Cells on coverslips were fixed in 4% paraformaldehyde for 20–30 min at room temperature. After three washes in PBS, cells were blocked in blocking buffer (PBS, 10% donkey serum, 0.1% Triton X-100) for 1 h at room temperature. Cells were incubated with primary antibodies at 4 °C overnight, washed three times with PBS and incubated with a secondary antibody for 1 h at room temperature. Then, cells were washed and stained with Hoechst 33258 (Sigma D9542) for 5–10 min. Primary antibodies used in this study were OCT4 (1:1000, Santa Cruz), SOX2 (1:1000, R&D System), PAX6 (1:1000, Covance), TUJ1 (1:1000, Sigma), Synapsin1 (1:1000, Sigma), NKX2.5 (1:1000, Santa Cruz), cTnT (1:500, Abcam), α-actinin (1:500, Sigma-Aldrich), MLC2a (1:500, Synapic systems), AFP (1:100, GeneTex) and ALB (1:100, GeneTex).

### Flow cytometry

Cells were dissociated into single cells with accutase at 37 °C for 3 min followed by fixation staining using the FACS Kit (BD bioscience) treatment according to the manufacturer’s instructions. Samples were sorted on a FACSVerse flow cytometer (BD Biosciences) and analyzed in Flowjo (v10). Antibodies used in this study were OCT4 (1:600, Santa Cruz), SOX2 (1:600, R&D System), PAX6 (1:600, DSHB), cTnT (1:500, Abcam), ALB (1:100, GeneTex), and isotype control mouse IgG (1:300, eBioscience).

### Mixed lymphocyte reactions

PBMCs used for MLR were obtained from healthy donors at Zhang Lab, Tongji University by centrifugation in Ficoll-Hypaque solution (MP Biomedicals) gradients. To assess PBMCs proliferation, PBMCs were labeled with CellTrace CFSE (Thermo Fisher Scientific). Neurons, cardiomyocytes and hepatocytes were pretreated with 25 ng/mL IFN-γ (PeproTech) for 48 h, then irradiated with 4000 rads before cocultured with CFSE-labeled PBMCs. 1 × 10^5^ neurons, cardiomyocytes and hepatocytes were, respectively, incubated with the PBMCs at a ratio of 1:1 in a 96-well round-bottom plate in RPMI-1640 supplemented with 2 mM L-glutamine, 5% heat-inactivated human AB serum (GEMINI Bio-products), 20 U/mL IL-2, and 50 μM β-mercaptoethanol for 6 days. PBMCs cultured with Phytohemagglutinin-L (PHA, Invitrogen) were used as a positive control. PBMCs were stained with anti-CD45 antibody (BD Bioscience) before CFSE intensity analysis on FACSVerse flow cytometer (BD Biosciences). Data were plotted using FlowJo software (v10).

### Teratoma formation

WT and hypoimmunogenic hPSCs were injected subcutaneously on the back of NSG mice (Shanghai MODEL ORGANISMS, China). After 2 months, mice with teratomas were sacrificed. Then, teratomas were stained with H&E. All animal experiments were conducted in accordance with the Guide for the Care and Use of Animals for Research Purposes and approved by the Tongji University Animal Care Committee.

### Periodic acid Schiff staining

Periodic acid Schiff (PAS) staining was performed by using the PAS staining kit (Abcam) according to the manufacturer’s instructions. Briefly, slides were immersed in PAS solution for 5–10 min, rinsed and immersed in Schiff's solution for 15–30 min. After washing, hematoxylin staining and bluing, the slides were incubated in Light Green Solution for 2 min followed by dehydration and mounting.

### Indocyanine green uptake and release

Indocyanine green (ICG) (Sigma) was dissolved in DMSO at 5 mg/mL. Cells were exposed to ICG diluted freshly in culture medium to 1 mg/mL for 30 min at 37 °C. After washing with PBS, cells were refilled with the culture medium and incubated for 6 h. The uptake and release of cellular ICG were examined under OLYMPUS microscope (OLYMPUS BX53).

### ALB secretion

The protein level of ALB in the culture medium was determined with an ELISA Kit (Elabscience) according to the manufacturer’s instructions. Briefly, 50 μL of samples was mixed with 50 μL of biotinylated detection Ab working solution and incubated for 90 min at 37 °C. After washing, 100 μL of HRP conjugate working solution was added and incubated for 30 min at 37 °C. The plate was washed followed by an incubation with 90 μL substrate reagent for 15 min at 37 °C. 50 μL stop solution was added immediately before reading the plate at 450 nm. Results were calculated according to standards.

### Statistical analyses

Data were presented as mean ± SEM. The statistical significance of differences was determined by unpaired two-tailed Student’s *t* test. *P* < 0.05 was considered statistically significant.

## Supplementary Information

**Additional file 1: Figure S1** Hypoimmunogenic hPSCs showed normal frequency of teratoma formation and expected genomic manipulations. **A** Genomic DNA PCR showing B2M knockout in B2M^null^ hPSCs and HLA-G insertion in B2M^mHLAG^ and B2M^m/sHLAG^ hPSCs at high passages. **B** HLA-G5 mRNA expression in hypoimmunogenic hPSCs compared to WT by qPCR. Data are represented as mean ± SEM. ***p < 0.001, *n* = 3, Student’s *t* test. **C** Frequency of teratoma formation of WT and hypoimmunogenic hPSCs. **Figure S2** Mixed lymphocyte reaction of hypoimmunogenic hPSCs-derived neurons, cardiomyocytes and hepatocytes. **A** HLA genotyping of donor PBMCs. Table shows HLA-I genotyping (A, B and C alleles). **B** Flow cytometry analysis showing percentages of CFSE-labeled allogeneic PBMCs (donor #01) with neurons, cardiomyocytes and hepatocytes derived from WT, B2M^null^, B2M^mHLAG^, and B2M^m/sHLAG^ hPSCs. PBMCs cultured with PHA were used as a positive control (PC). PBMCs cultured only were used as a negative control (NC).

**Additional file 2: Video S1.** Beating cardiomyocytes derived from WT hPSCs.

**Additional file 3: Video S2.** Beating cardiomyocytes derived from B2M^null^ hPSCs.

**Additional file 4: Video S3.** Beating cardiomyocytes derived from B2M^mHLAG^ hPSCs.

**Additional file 5: Video S4.** Beating cardiomyocytes derived from B2M^m/sHLAG^ hPSCs.

## Data Availability

All data generated or analyzed during this study are included in this published article. The raw sequence data reported in this paper have been deposited in the Genome Sequence Archive (Genomics, Proteomics & Bioinformatics 2021) in the National Genomics Data Center (Nucleic Acids Res 2021), China National Center for Bioinformation/Beijing Institute of Genomics, Chinese Academy of Sciences, under accession number HRA001756 at https://ngdc.cncb.ac.cn/gsa-human.

## Abbreviations

hPSCs: Human pluripotent stem cells
HLA: Human leukocyte antigen
CTLA4: Cytotoxic T lymphocyte antigen 4
PD-L1: Programmed death ligand-1
MHC: Major histocompatibility complex
NSG: NOD scid gamma mouse
H&E: Hematoxylin–eosin
AP: Action potential
RMP: Resting membrane potential
cTnT: Cardiac troponin T
APD: Action potential duration
APA: Action potential amplitude
ICG: Indocyanine green
PAS: Periodic acid Schiff
