# Supplementary figures and images for: Hypoimmunogenic human pluripotent stem cells are valid cell sources for cell therapeutics with normal self-renewal and multilineage differentiation capacity
Source: Stem Cell Res Ther. 2023 Jan 24;14:11. doi: 10.1186/s13287-022-03233-z (PMC9872349; doi:10.1186/s13287-022-03233-z)

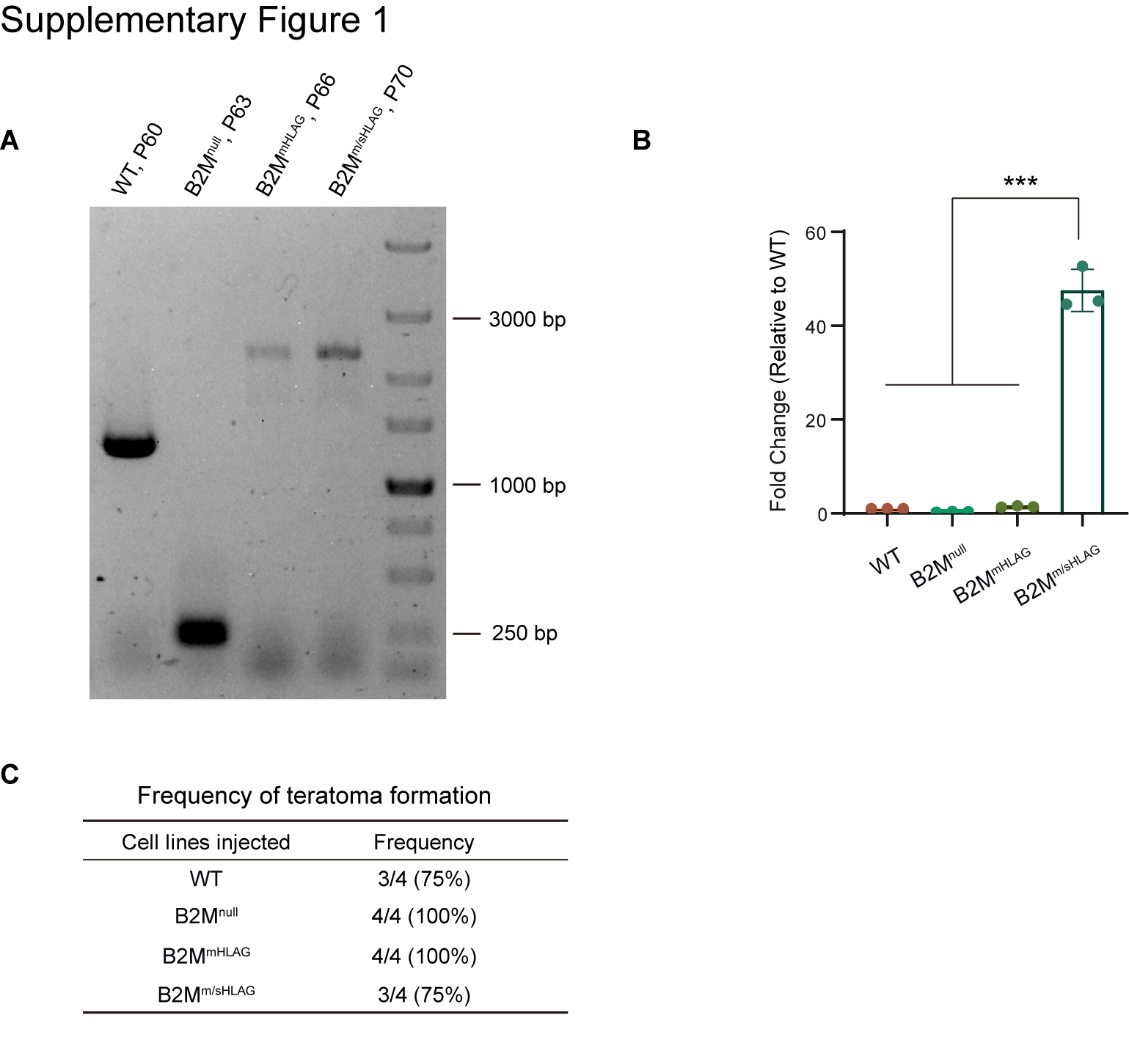


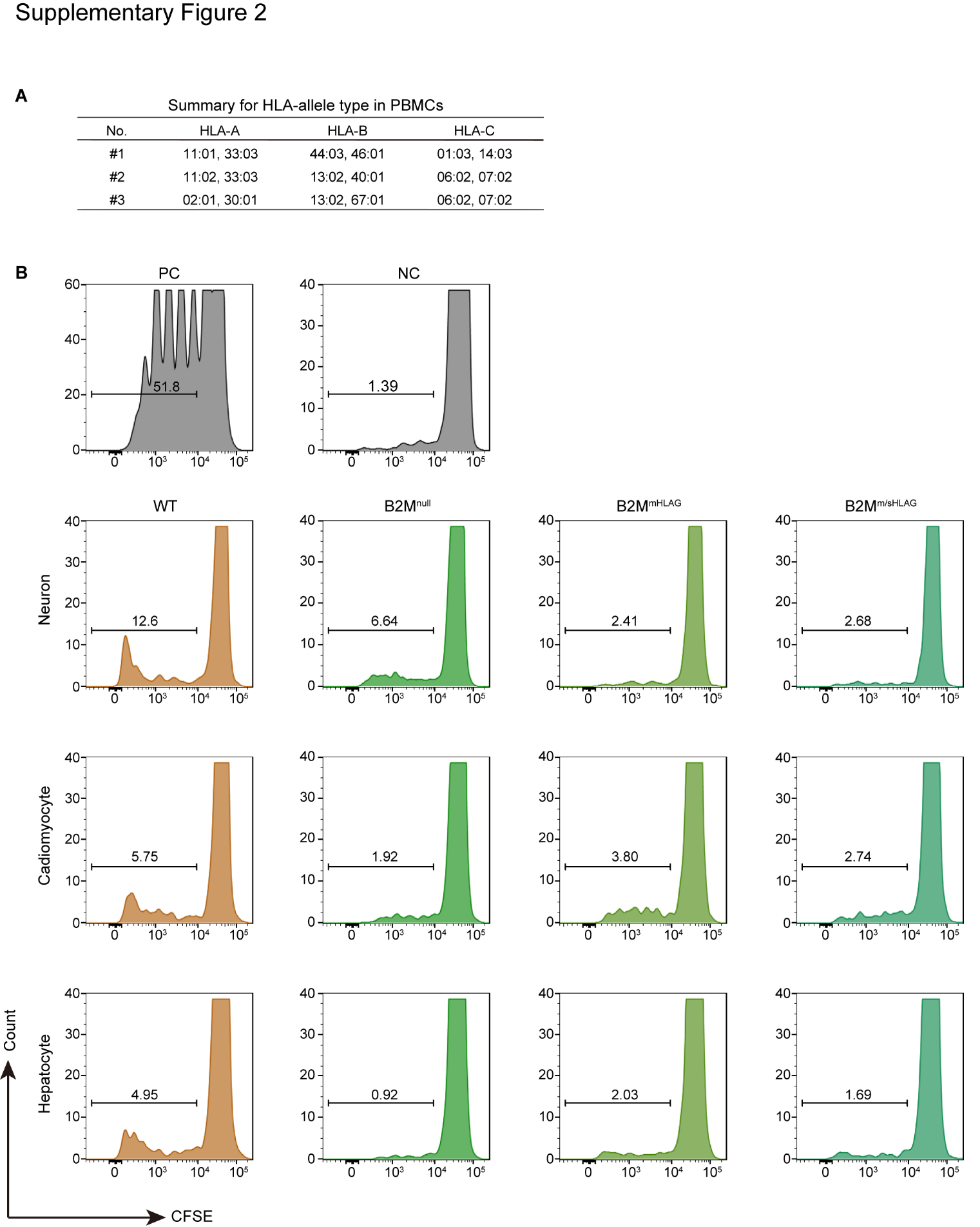

Supplement: Supplementary file 1 — Additional file 1: Figure S1 Hypoimmunogenic hPSCs showed normal frequency of teratoma formation and expected genomic manipulations. A Genomic DNA PCR showing B2M knockout in B2Mnull hPSCs and HLA-G insertion in B2MmHLAG and B2Mm/sHLAG hPSCs at high passages. B HLA-G5 mRNA expression in hypoimmunogenic hPSCs compared to WT by qPCR. Data are represented as mean ± SEM. ***p < 0.001, n = 3, Student’s t test. C Frequency of teratoma formation of WT and hypoimmunogenic hPSCs. Figure S2 Mixed lymphocyte reaction of hypoimmunogenic hPSCs-derived neurons, cardiomyocytes and hepatocytes. A HLA genotyping of donor PBMCs. Table shows HLA-I genotyping (A, B and C alleles). B Flow cytometry analysis showing percentages of CFSE-labeled allogeneic PBMCs (donor #01) with neurons, cardiomyocytes and hepatocytes derived from WT, B2Mnull, B2MmHLAG, and B2Mm/sHLAG hPSCs. PBMCs cultured with PHA were used as a positive control (PC). PBMCs cultured only were used as a negative control (NC). [file 13287_2022_3233_MOESM1_ESM.docx]
